# MED12 exon 2 mutations are common in uterine leiomyomas from South African patients

**DOI:** 10.18632/oncotarget.370

**Published:** 2011-12-19

**Authors:** Netta Mäkinen, Hanna-Riikka Heinonen, Shane Moore, Ian P.M. Tomlinson, Zephne M. van der Spuy, Lauri A. Aaltonen

**Affiliations:** ^1^ Department of Medical Genetics, Genome-Scale Biology Research Program, University of Helsinki, Helsinki, Finland; ^2^ Department of Obstetrics and Gynaecology, Faculty of Health Sciences, University of Cape Town, South Africa; ^3^ Wellcome Trust Centre for Human Genetics and NIHR Comprehensive Biomedical Research Centre, Nuffield Department of Clinical Medicine, Roosevelt Drive, University of Oxford, Oxford OX3 7BN, UK

**Keywords:** Uterine leiomyoma, fibroid, MED12, ethnicity

## Abstract

Uterine leiomyomas, or fibroids, are extremely common tumors. Regardless of their benign nature, fibroids can cause considerable morbidity. Women with African ancestry have a threefold increased risk of developing uterine leiomyomas with a greater symptom severity when compared to white women. Recently, we demonstrated that exon 2 of the *MED12* gene is somatically altered in up to 70 per cent of uterine leiomyomas in a series of Finnish (Caucasian) patients. To validate these results in other populations, we sequenced a set of 28 uterine leiomyomas for *MED12* exon 2 mutations from 18 different Black African or Coloured South African patients. We observed 14 mutation positive lesions (50%). When corrected by tumor size, these results are very similar to those derived in the Finnish material. This study confirms a major role of MED12 in the genesis of leiomyomas, regardless of ethnicity.

## INTRODUCTION

Uterine leiomyomas, also known as fibroids, are benign tumors for which the lifetime risk in women over the age of 45 has been estimated to exceed 60% [1]. Fibroids arise from the smooth muscle cells of the myometrium and can cause significant morbidity, such as abnormal uterine bleeding, abdominal pain and discomfort, pregnancy complications and even infertility [2]. Fibroids are the most common cause for hysterectomy, and they have a considerable socio-economic impact [3-4].

Uterine leiomyomas are monoclonal, oestrogen and progesterone dependent tumors, which occur in women of reproductive age, and typically regress with the onset of menopause. On the other hand, parity and use of oral contraceptives have been suggested to protect women from the development of fibroids [1]. It is also known that uterine leiomyomas do not affect all ethnicities equally. Women with African ancestry have a threefold risk of developing uterine leiomyomas compared with white women [5], and Africans have also been reported to have an earlier age at onset with larger, more numerous, and more rapidly-growing fibroids [6-7]. In addition to ethnicity, other factors, such as family history, smoking, alcohol intake, hypertension and increased body weight have been proposed to increase the risk of developing uterine leiomyomas [6, 8-10].

We recently identified various somatic mutations in exon 2 of the *mediator complex subunit 12* (*MED12*) gene, in as many as 70% of the studied uterine leiomyomas obtained from patients of Finnish (Caucasian) origin [11]. The mutation hot spot affected an evolutionary conserved region of the MED12 protein, and according to our results, large fibroids tended to have slightly fewer mutations than small fibroids. MED12 is part of the 26-subunit Mediator complex which is thought to regulate global as well as gene-specific transcription by bridging distant regulatory DNA elements to the RNA polymerase II initiation complex [12].

The aim of this study was to investigate the frequency of *MED12* exon 2 mutations in uterine leiomyomas of South African patients to confirm that MED12 has a major role in the genesis of this tumor type in populations other than Finns. Thus, we screened a total of 28 uterine fibroids from 18 individual patients for these mutations.

## RESULTS

We sequenced a set of 28 uterine leiomyomas from 18 South African patients. Fourteen leiomyomas (50%) harbored a mutation in *MED12* exon 2 (Table 1). Eight of these mutations were located in codon 44. In addition, two fibroids (7%) displayed a missense mutation in codon 36 and one fibroid (3.6%) in codon 43. We also observed two (7%) exonic insertion-deletion type mutations and one somatic intronic T to A mutation (3.6%) eight base pairs upstream of the splice acceptor site of exon 2. All three mutations are predicted to result in an in-frame transcript. The somatic nature of the mutations was verified in all cases where normal tissue DNA was available (nine). Nine patients did not have any mutations in *MED12* exon 2.

**Table 1:** Patient information and *MED12* exon 2 mutation status of the studied fibroids

Patient	Age at Diagnosis	Ethnicity	Number of Fibroids	Fibroid	Size	Status of *MED12* exon 2	Myometrium
**FG106**	50	Black South African	3	FG106_1	Fundal 20 × 10 cm	c.130G>A, p.G44S	x
				FG106_2	Anterior 15 × 15 cm	c.100-8T>A, p.E33_D34insPQ	
**FG107**	48	Coloured	Multiple	FG107_1	Posterior 3.7 × 4.0 cm	c.107T>G, p.L36R	x
**FG108**	41	Coloured	Not reported	FG108_1	4.4 × 4.2 cm	wt	x
				FG108_2	2.5 × 3.3 cm	c.131G>A, p.G44D	
				FG108_3	Not reported	c.149_163del15, p.A50_D54del	
**FG109**	46	Coloured	Multiple	FG109_1	Not reported	wt	x
				FG109_2	Not reported	wt	
**FG141**	27	Black South African	15	FG141_1	Fundal no size	c.130G>C, p.G44R	
				FG141_3	Lateral no size	c.131G>A, p.G44D	
**FG142**	59	Black South African	Multiple	FG142_2	Lateral no size	c.107T>G, p.L36R	x
**FG146**	39	Coloured	1	FG146_1	7.0 × 7.1 cm	wt	x
**FG147**	30	Coloured	2	FG147_1	Anterior 6.7 × 6.7cm	wt	
				FG147_2	Posterior no size	c.130G>T, p.G44C	
**FG149**	48	Coloured	2	FG149_1	1.3 × 1.3 cm	wt	x
**FG150**	47	Coloured	2	FG150_1	Anterior 4.3 × 4.1 cm	wt	x
				FG150_2	Posterior 4.5 × 5.0 cm	wt	
**FG151**	39	Coloured	2	FG151_1	Anterior no size	wt	
				FG151_2	Posterior no size	wt	
**FG152**	41	Coloured	2	FG152_1	Anterior 7.3 × 5.9 cm	c.131G>C, p.G44A	x
				FG152_2	Posterior 11.0 × 7.5 cm	c.131G>A, p.G44D	
**FG153**	50	Coloured	2	FG153_1	Anterior no size	c.128A>C, p.Q43P	
**FG154**	45	Black South African	1	FG154_1	Inferior 5.0 × 4.5 cm	wt	x
**FG155**	33	Coloured	Multiple	FG155_1	Lateral no size	c.122_148del27, p.V41_P49	x
				FG155_2	Lateral no size	c.131G>A, p.G44D	
**FG157**	32	Black South African	Multiple	FG157_1	Not reported	wt	x
**FG166**	48	Coloured	Multiple	FG166_1	Anterior no size	wt	x
**FG169**	41	Black South African	2	FG169_1	Posterior 17.8 × 11.5 cm	wt	x

The difference between the frequency of mutation positive fibroids in women with mixed ancestry (Coloured) and Black South African women was statistically significant when compared with the frequency of mutation positive lesions in Finnish (Caucasian) women (p-value = 0.045) [11]. However, if corrected by tumor size, the results in the two series were very similar (p-value = 0.69).

## DISCUSSION

To our knowledge, this is the first description of *MED12* exon 2 mutation analysis in uterine leiomyomas from other than white women. In this study, a set of 28 uterine leiomyomas from 18 South African patients was sequenced for *MED12* exon 2 mutations to study the role of MED12 in tumorigenesis of fibroids also in other populations than Finns. Altogether, 14 (50%) mutation positive lesions were observed.

Ethnicity is an important epidemiological risk factor for uterine leiomyomas in the general population. The effect of race on incidence and severity of fibroids is particularly significant. Several studies have reported a higher incidence of fibroids among Black women than other racial and ethnic groups including Caucasian, Hispanic, and Asian women [6, 10, 13]. Moreover, Black women tend to have more severe disease than Caucasian women, including an earlier age at diagnosis and at hysterectomy, with larger, more numerous, and more rapidly growing fibroids [6-7, 13].

The reasons for ethnic variation in uterine leiomyoma occurrence are unknown. Various possible causes for higher prevalence and greater symptom severity among Black women have been proposed. For example, the differences may be due to genes that confer increased risk for poor outcome. Uterine leiomyomas are hormonally responsive tumors, and for instance, Black women have been reported to have a higher prevalence of oestrogen receptor-α PP variant than white women [14]. The variant has been associated with an increased risk of uterine leiomyomas in both ethnicities.

Our studies demonstrate that fibroids from both Caucasians as well as women with African descent frequently harbor mutations in *MED12* exon 2. The South African series displayed significantly fewer mutation-positive lesions than the previously published Finnish series. However, the tumors in the South African series tended to be larger, and because the results in the Finnish series had indicated an inverse correlation between *MED12* mutations and tumor size, we analyzed the results after correction of this tumor characteristic. Indeed, after this correction the results were very similar. While further work remains to be done to clarify the reasons behind the observed differences, this study confirms a major role of MED12 in the tumorigenesis of leiomyomas, regardless of ethnicity.

## MATERIALS AND METHODS

### Patient Material

DNA of 28 uterine leiomyoma and 14 myometrium samples was obtained from the Department of Obstetrics and Gynaecology, Faculty of Health Sciences, University of Cape Town, South Africa. DNA had been extracted from fresh frozen tissue samples. Altogether 18 individual patients were included to this study and from each patient 1-3 uterine leiomyomas were examined (Table 1). The patient series comprised of twelve women with mixed ancestry (Coloured) and six Black South African women. This study was approved by the local Human Research Ethics Committee (REF: 008/1995 and REF: 433/2011).

### Amplification of *MED12* exon 2

Using previously reported primer sequences [11] the desired DNA fragment was amplified with AmpliTaqGold® enzyme (Applied Biosystems, Foster City, CA, USA). The PCR products were purified using ExoSAP-IT PCR Purification Kit (USB Corporation, Cleveland, OH, USA) and the sequencing reactions were performed utilizing the Big Dye Terminator v.3.1 Kit (Applied Biosystems, Foster City, CA, USA) according to the manufacturer's instructions. Sequencing was performed on an ABI3730 Automatic DNA Sequencer (Applied Biosystems at FIMM Genome and Technology Centre Finland). The sequence graphs were analyzed both manually and on computer with Mutation Surveyor - program (Softgenetics, State College, PA, USA).

### Statistical Analysis

Statistical analyses were performed using R software, version 2.14.0. Differences between the proportion of mutation positive lesions in uterine leiomyomas with Finnish and South African patients were undertaken with Pearson's χ^2^ test with 1 *df*. Also the differences between the frequency of large (at least 5.5cm diameter) mutation positive lesions in the South African series versus the Finnish series, was evaluated with Fisher's exact test.
